# TNF-α Induced Myotube Atrophy in C2C12 Cell Line Uncovers Putative Inflammatory-Related lncRNAs Mediating Muscle Wasting

**DOI:** 10.3390/ijms23073878

**Published:** 2022-03-31

**Authors:** Tomasz Powrózek, Dominika Pigoń-Zając, Marcin Mazurek, Michael Ochieng Otieno, Mansur Rahnama-Hezavah, Teresa Małecka-Massalska

**Affiliations:** 1 Department of Human Physiology, Medical University of Lublin, 20-080 Lublin, Poland; pigon.dominika@gmail.com (D.P.-Z.); marcinmazurek1212@gmail.com (M.M.); tmalecka@gmail.com (T.M.-M.); 2 Haematological Malignancies H12O Clinical Research Unit, Spanish National Cancer Research Centre, 28029 Madrid, Spain; michaelochieng@yaho.com; 3 Chair and Department of Dental Surgery, Medical University of Lublin, 20-093 Lublin, Poland; mansur.rahnama@umlub.pl

**Keywords:** muscle atrophy, inflammation, TNF-α, lncRNA

## Abstract

Background: Muscle atrophy is a complex catabolic condition developing under different inflammatory-related systemic diseases resulting in wasting of muscle tissue. While the knowledge of the molecular background of muscle atrophy has developed in recent years, how the atrophic conditions affect the long non-coding RNA (lncRNAs) machinery and the exact participation of the latter in the mediation of muscle loss are still unknown. The purpose of the study was to assess how inflammatory condition developing under the tumor necrosis factor alpha (TNF-α) treatment affects the lncRNAs’ expression in a mouse skeletal muscle cell line. Materials and method: A C2C12 mouse myoblast cell line was treated with TNF-α to develop atrophy, and inflammatory-related lncRNAs mediating muscle loss were identified. Bioinformatics was used to validate and analyze the discovered lncRNAs. The differences in their expression under different TNF-α concentrations and treatment times were investigated. Results: Five lncRNAs were identified in a discovery set as atrophy related and then validated. Three lncRNAs, Gm4117, Ccdc41os1, and 5830418P13Rik, were selected as being significant for inflammatory-related myotube atrophy. Dynamics changes in the expression of lncRNAs depended on both TNF-α concentration and treatment time. Bioinformatics analysis revealed the mRNA and miRNA target for selected lncRNAs and their putative involvement in the molecular processes related to muscle atrophy. Conclusions: The inflammatory condition developing in the myotube under the TNF-α treatment affects the alteration of lncRNAs’ expression pattern. Experimental and bioinformatics testing suggested the prospective role of lncRNAs in the mediation of muscle loss under an inflammatory state.

## 1. Introduction

Skeletal muscle atrophy is a complex process resulting from an imbalance between anabolic and catabolic conditions. It is characterized by a decreased protein synthesis and enhanced proteolysis within the myocyte particularly caused by the hyperactivation of major cellular degradation pathways, including the ubiquitin-proteasome and autophagy-lysosomal systems [1,2,3]. The above mentioned conditions are the emaciated consequences of different systemic disorders, including cancer, chronic diseases, starvation, myopathies, and aging [4,5].

The pathogenesis of muscle atrophy is composite, but the corresponding hyperactivation of a systemic inflammatory response is a frequent event observed during chronic diseases. Thus, the elevation of pro-inflammatory cytokines’ level is thought to play an apparent role for muscle wasting [6]. In particular, tumor necrosis factor-α (TNF-α) has been shown to directly promote muscle loss through the activation of the nuclear factor-κB (NF-κB) pathway and the activation of the both ubiquitin-dependent proteolysis and myotube apoptosis [7,8]. The TNF-α-mediated activation of the NF-κB pathway leads to the proteolysis of specific myocyte proteins and an increase in expression of other pro-inflammatory cytokines, such as IL-1 and IL-6, which activate muscle wasting upon the inflammatory state [9,10,11]. Moreover, the upregulation of NF-κB signaling significantly reduces the expression of muscle differentiation-related genes, mainly myoblast determination protein 1(MyoD) and myogenin (MYOG), making muscle regeneration impracticable [12].

The knowledge of muscle atrophy and its molecular background has developed in recent years. However, how the atrophic conditions affect the non-coding RNA (ncRNA) machinery and the exact participation of the regulatory network of the latter in the mediation of muscle wasting are still unknown. Among the ncRNAs, a group of molecules longer than 200 bases in length, called long non-coding RNAs (lncRNAs), seems to play an important role in the regulation of gene expression and management of cellular processes via different mechanisms, such as miRNA sponging or interaction with DNA and proteins. Even though lncRNAs do not encode proteins, their transcripts modulate diverse cellular processes, including cell differentiation, apoptosis, and autophagy [13,14].

According to recent findings, some lncRNAs are required for skeletal muscle formation and terminal differentiation of myoblasts, such as SRA and MUNC [15]. The lncRNA, such as MyoD and IRS1, regulates the expression of the genes involved in cell proliferation and muscle differentiation; MD1, DUM, and MALAT1 regulate myogenesis [15,16,17,18]. Although particular lncRNAs were identified as factors regulating muscle development and viability, little is known about their exact role in the muscle atrophy machinery. Until now, the changes in the expression of lncRNAs in the muscle cell under the inflammatory state remain unclear. Thus, it is difficult to predict their unique role in atrophy. The purpose of the study was to assess how an inflammatory condition developed under TNF-α stimulation affects the lncRNAs’ expression in a mouse skeletal muscle cell line. Based on the study results, some new lncRNAs were discovered as being important for the process of inflammatory-related muscle atrophy.

## 2. Results

### 2.1. Atrophy Model

**Figure 1:**
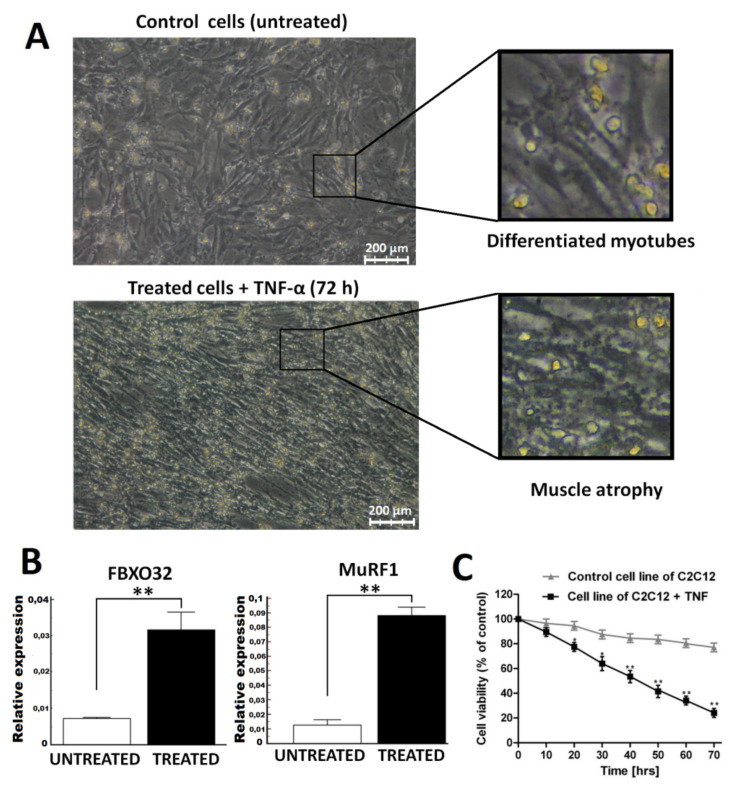
C2C12 myotube atrophy model: (**A**) Visible morphologic differences between C2C12 cell culture treated with 100 ng/mL TNF-α after 72 h (atrophy) and untreated cells (well-differentiated myotubes); (**B**) differences in expression of muscle atrophy-related markers between TNF-α-treated (atrophy) and control cells (untreated); (**C**) differences in cell viability between cell line treated with TNF-α and control cells (* *p* < 0.05 and ** *p* < 0.001 compared to control cells).

In Figure 1, we illustrate the differences in myotube morphology, expression of atrophy markers, and myotube viability between a C2C12 cell line treated with TNF-α for 72 h and untreated cells (control). Figure 1A represents the morphological differences between myotube atrophy and control cells. Figure 1B illustrates significant differences in FBXO32 and MuRF1 expression between the studied cultures. In Figure 1C, the differences in cell viability aredemonstrated for control and treated C2C12 cells.

### 2.2. DElncRNA

**Figure 2:**
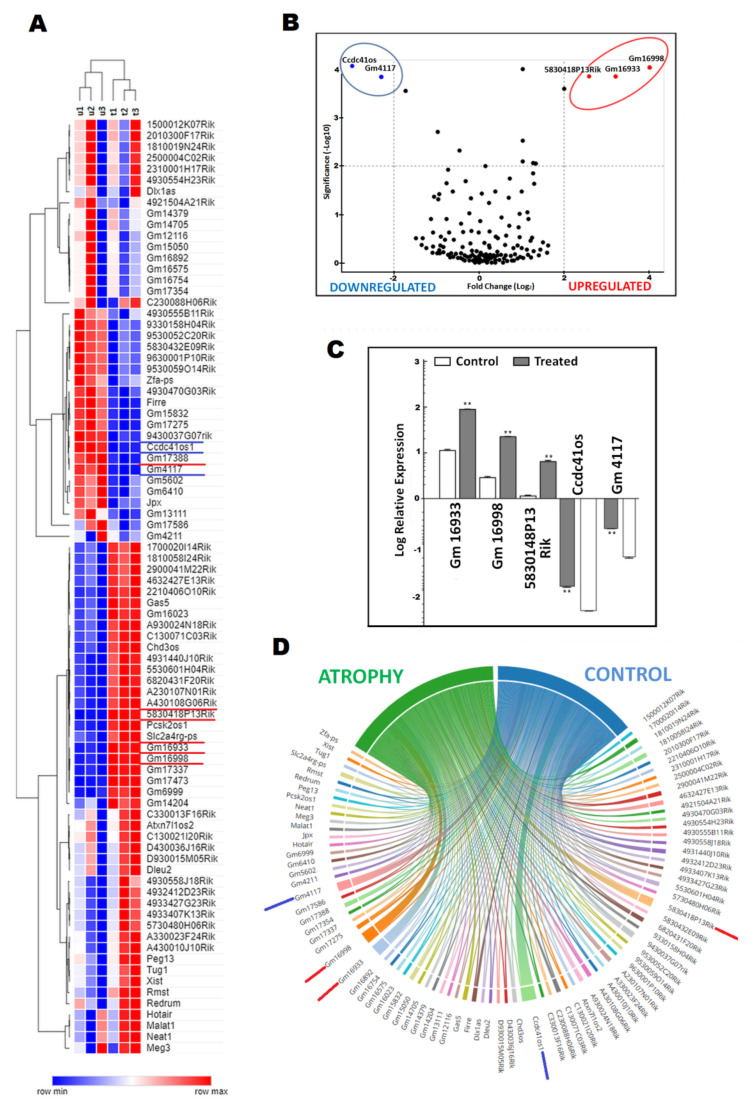
Identification of DElncRNAs between C2C12 myotube atrophy and control C2C12 cells in a discovery set: (**A**) heat map of 84 lncRNAs demonstrating at least a 2-fold expression difference between samples; (**B**) volcano plot illustrating differences in expression of 176 lncRNAs between samples as well as the top significant DElncRNAs; (**C**) expression differences of the top five DElncRNAs between atrophic C2C12 myotube culture and control cells; (**D**) chord diagram illustrating connections/relationship between lncRNAs demonstrating at least a 2-fold expression difference and either atrophic or non-atrophic conditions in C2C12 myotube culture. (** *p* < 0.001 compared to control cells).

After incubation, cells were harvested both from treated and untreated C2C12 cell cultures for RNA extraction. In a discovery set, cells were harvested both from a culture incubated with 100 ng/mL TNF-α and an untreated culture after 72 h. In a discovery set, a total of 73 (41.4%) lncRNAs were downregulated and 103 (58.6%) were upregulated in atrophy C2C12 myotubes treated with TNF-α comparing with control cells. The generated heat map illustrating DElncRNA(only 84 lncRNAs demonstrating at least 2-fold difference in expression between treated and untreated cells) with a hierarchical clustering between three untreated and three cell lines treated with TNF-α is demonstrated in Figure 2A. The most biologically significant DElncRNA (top DElncRNA) among all of the studied samples was revealed by volcano plot analysis. It enabled the visual identification of DElncRNA with large fold changes that werealso statistically significant (Figure 2B). Adjusting the fold differences and p values between the samples, the top five DElncRNA were selected as atrophy-related, and they were as follows: Gm4117 (ENSMUSG00000089940), Ccdc41os1 (Cep83os; ENSMUSG00000097164), Gm16998 (ENSMUSG00000097069), Gm16933 (ENSMUSG00000086289), and 5830418P13Rik (ENSMUSG00000086236). Gm4117 and Ccdc41os1 were downregulated, and the other three DElncRNA were upregulated between untreated and atrophic cells. In Figure 2C, we demonstrate the log fold change in the expression levels between control and treated C2C12 cells. We found decreases in the expression levels of Gm4117 and Ccdc41os1 of 4.1fold and 8.2fold, respectively. On the contrary, Gm16998, Gm16933, and 5830418P13Rik demonstrated an increase in expression of 8fold, 8.2fold, and 4.7fold, respectively, in contrast to the control cells (all *p* < 0.001). Constructing chord diagrams, we visualized the network of the contribution between DElncRNA and the atrophy and non-atrophyclusters (Figure 2D). Additionally, we derived the top DElncRNA and visualized their relationship between clusters (the size of the chords demonstrates the differences in the strength of the connection between DElncRNA and the clusters).

### 2.3. Changes in the Expression of the Top DElncRNA under Different Treatment Conditions

**Figure 3:**
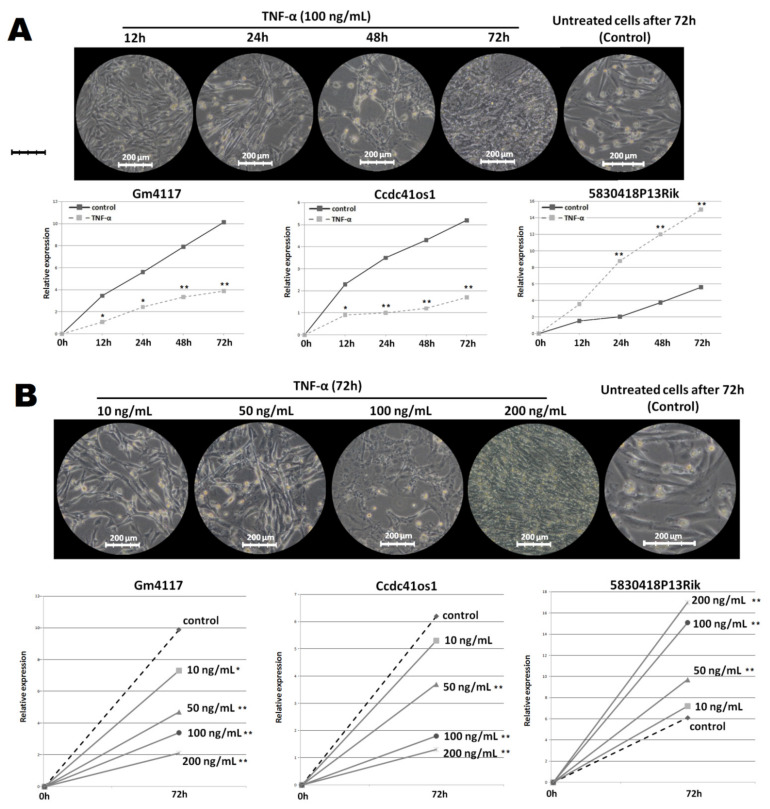
Dynamics changes in the expression of identified DElncRNAs under different TNF-α treatment conditions: (**A**) impact of 100 ng/mL TNF-α on myotube morphology within different treatment times (12, 24, 28, and 72 h) and corresponding dynamics changes in expression of DElncRNAs; (**B**) changes in myotube morphology 72 h after treatment with different TNF-α concentrations (10, 50, 100, and 200 ng/mL) and corresponding differences in expression of DElncRNAs (* *p* < 0.05; ** *p* < 0.001).

Top DElncRNAs identified in a discovery set were independently validated by qRT-PCR under different TNF-α treatment conditions in a new set of C2C12 myotube cell cultures (Supplementary Figure S1). In separate and independent experiments, three out of five selected DElncRNAs (Gm4117, Ccdc41os1, and 5830418P13Rik) demonstrated significant differences in expression between a C2C12 myotube atrophy culture and control cells. For this reason, the expression of Gm4117, Ccdc41os1, and 5830418P13Rik was analyzed in C2C12 cell cultures under different TNF-α concentrations and treatment times (Figure 3). First, we observed dynamics changes in expression after 12, 24, 48, and 72 h of three lncRNAs in C2C12 cells treated with 100 ng/mL of TNF-α and in non-treated C2C12 cells. The most significant differences in expression of all DElncRNAs between the control and treated cells were observed after 48 and 72 h of TNF-α treatment (*p* < 0.001) (Figure 1A). Second, we investigated the impact of various TNF-α concentrations (10, 50, 100, and 200 ng/mL) on DElncRNAs’ expression dynamics 72 h after treatment, comparing with untreated cells. A significant relationship between TNF-α concentrations and the expression of DElncRNAs was noticed. While a gradual increase in TNF-α concentration resulted inthe gradual decrease in the expression of Gm4117 and Ccdc41os1, theexpression of 5830418P13Rik was elevated compared to untreated C2C12 cells. The most significant differences in DElncRNAs’ expression between cell cultures were observed with 50, 100, and 200 ng/mL TNF-α (*p* < 0.001) (Figure 3B).

### 2.4. Construction of the Interaction Network between Top DElncRNA-mRNA and miRNA; Functional Annotation of Key DElncRNA, miRNAs, and PPI Network

Top DElncRNAs were screened in order to discover a regulatory network and interaction with mRNA and miRNA with the application of GO and KEGG. First, we predicted the mRNA targets for the top DElncRNA with the aforementioned bioinformatics tools. Thereafter, the regulatory PPI network was constructed with the use of String and ShinyGO. Regarding the sponging ability of the top DElncRNA for miRNA, the role of the predicted small ncRNAs was investigated in the signaling pathways.

### 2.5. Gm4117: ENSMUSG00000089940

**Figure 4:**
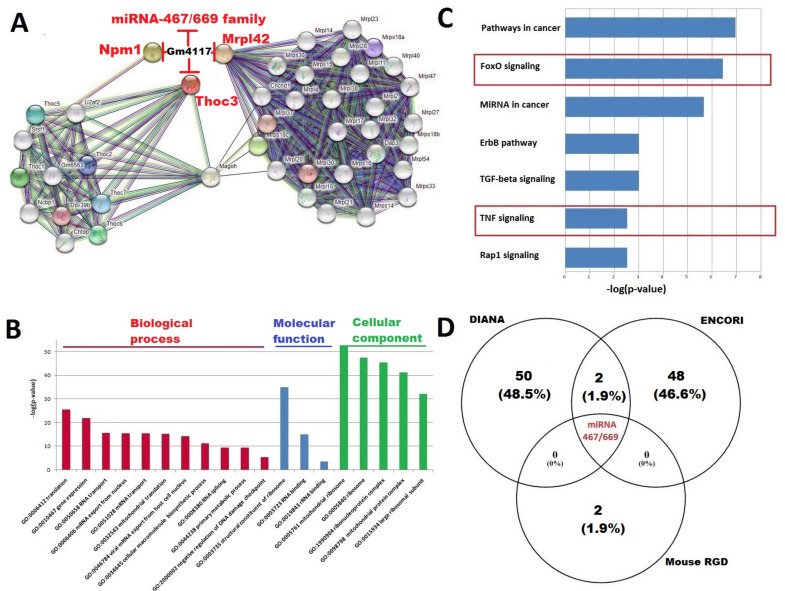
Bioinformatics analysis for Gm4117: (**A**) constructed protein–protein interaction (PPI) network and predicted miRNA-467/669 family interacting with Gm4117; (**B**) result of GO enrichment analysis and top, most significantly enriched terms for Npm1,Thoc3, and Mrpl42; (**C**) result of KEGG enrichment analysis and top, most significantly enriched terms for Npm1,Thoc3, and Mrpl42 (the key pathways related to muscle atrophy were marked); (**D**) Venn diagram illustrating the compatibility between miRNA prediction by different databases.

Data derived from bioinformatics software predicted the mRNA of Npm1 (nucleophosmin 1), Thoc3 (THO complex 3), and Mrpl42 (mitochondrial ribosomal protein L42) proteins as targets for Gm4117. Based on the prediction score, the PPI network was constructed. The PPI network that interacted with Gm4117 consisted of 43 nodes and 489 edges (enrichment *p*-value <1.0 × 10^−16^) (Figure 4A). According to the GO enrichment analysis, the translation (false discovery rate (FDR) = 2.56 × 10^−26^), gene expression (FDR = 1.48 × 10^−22^), and RNA transport (FDR = 2.07 × 10^−16^) werethe most enriched GO terms related to the biological processes, the structural constituent of ribosome (FDR = 1.03 × 10^−35^) for molecular function and mitochondrial ribosome (FDR = 3.13 × 10^−53^), ribosome (FDR = 3.43 × 10^−48^), and ribonucleoprotein complex (FDR = 2.56 × 10^−46^) for the cellular component. The top, most significantly enriched GO terms including biological process, molecular function, and cellular component arepresented in Figure 4B. According to the KEGG enrichment analysis, ribosome (FDR =8.29 × 10^−28^), RNA transport (FDR = 1.61 × 10^−10^), and spliceosome (FDR = 6.08 × 10^−10^) were three significantly enriched pathways (Figure 4C). Using miRNAprediction tools, approximately 100 miRNAs were derived aspotential sponging targets for Gm4117. Applying a Venn diagram, the compatibility between databases predicting miRNA isdisplayed in Figure 4D. Each individual circle in the Venn diagram represents a database with the predicted number of miRNAs targeting individual lncRNA. Only the miRNA-467/669 family sequences were derived from studied databases as significant for the Gm4117 interaction network. Gm4117 contains sites formiRNA-467 (a,g,h,f) and miRNA-699 (a,g,i,j,n), the nine binding sites predicted in total(all demonstrate 6–8mer). Using DIANA-mirPath v3.0, the top predicted KEGG enrichment analysis scores for the miRNA-467/669 family were pathways in cancer (FDR = 1.10 × 10^−7^) and miRNA in cancer (FDR = 2.15 × 10^−6^). Interestingly, KEGG also emerged for FoxO signaling (FDR = 3.63 × 10^−7^) and TNF signaling (FDR = 3.0 × 10^−3^) as significantly enriched pathways interacting with the miRNA-467/669 family.

### 2.6. Ccdc41os1/Cep83os: ENSMUSG00000097164

**Figure 5:**
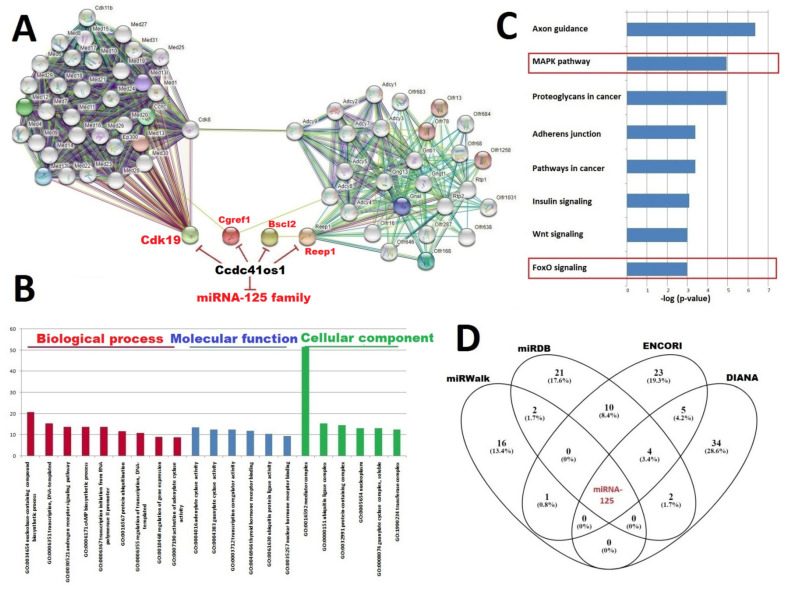
Bioinformatics analysis for Ccdc41os1: (**A**) constructed protein–protein interaction (PPI) network and predicted miRNA-125 interacting with Ccdc41os1; (**B**) result of GO enrichment analysis and top, most significantly enriched terms for predicted genes; (**C**) result of KEGG enrichment analysis and top, most significantly enriched terms for predicted genes (the key pathways related to muscle atrophy were marked); (**D**) Venn diagram illustrating the compatibility between miRNA prediction by different databases.

Ccdc41os1 contributes to the regulation of the network between Cdk19 (cyclin-dependent kinase 19), Cgref1 (cell growth regulator with EF-hand domain 1), Bscl2 (lipid droplet biogenesis-associated, seipin), and Reep1 (receptor accessory protein 1). The constructed PPI network consisted of 54 nodes and 648 edges (enrichment *p*-value < 1.0 × 10^−16^) (Figure 5A). The most enriched GO terms related to biological processes were nucleobase-containing compound biosynthetic process (FDR = 1.89 × 10^−21^) and transcription DNA-templated (FDR = 4.89 × 10^−16^); adenylate cyclase activity (FDR = 2.22 × 10^−14^) was a top GO term related to molecular function. Regarding the cellular component, the mediator complex (FDR = 2.35 × 10^−52^) and ubiquitin ligase complex (FDR = 4.15 × 10^−16^) were the most significantly enriched GO terms. A summary of the top GO enrichment terms is shown in Figure 5B. According to the KEGG enrichment analysis, the thyroid hormone signaling pathway (FDR = 3.32 × 10^−16^) and GABAergic synapse (FDR = 3.17 × 10^−9^) were significantly enriched pathways (Figure 5C). A total of 119 miRNAs were predicted for Ccdc41os1 as targets for all databases; however, only one miRNAfamily, miRNA-125, was unique for all databases (Figure 5D). Ccdc41os1 contains three binding sites for miRNA-125 (a,b,c) (all demonstrate 6–8mer). Each individual circle in the Venn diagram represents a database with the predicted number of miRNAs targeting individual lncRNA. Using DIANA-mirPath v3.0, the top predicted KEGG enrichment analysis scores for the miRNA-125 family were axon guidance (FDR = 4.23 × 10^−7^), MAPK pathway (FDR = 1.1 × 10^−5^), and proteoglycans in cancer (FDR = 1.1 × 10^−5^).

### 2.7. 5830418P13Rik: ENSMUSG00000086236

According to the DIANA–LncBase Predicted v2 tool, 5830418P13Rik is especially capable to target the miRNA-466 and -669 families (all predicted scores over 0.790). A direct analysis of the lncRNA targets did not reveal significantly affected mRNAs. Considering the 5830418P13Rik miRNAs’ regulatory network, the top GO and KEGG enrichment terms were indentified. The top GO terms related to molecular function were cell differentiation (FDR = 5.20 × 10^−30^) and cellular protein modification process (FDR = 1.85 × 10^−23^), whereas response to stress (FDR = 7.87 × 10^−3^) and catabolic process (FDR = 1.93 × 10^−11^) were highly related terms for biological function. As for the cellular component, chromosome organization (FDR = 2.98 × 10^−17^) was the most significantly enriched GO term. Regarding KEGG terms, pantothenate and CoA biosynthesis (FDR = 2.29 × 10^−3^), central carbon metabolism in cancer (FDR = 5.90 × 10^−3^), and lysine degradation (FDR = 6.88 × 10^−3^) were significant terms for this analysis.

## 3. Discussion

Muscle activity is influenced by different quality systems, which regulate the function of contractile proteins and, upon atrophic conditions, such as aging, inflammation, cancer, cachexia, and denervation, deregulate. However, an atrophic condition developing in a muscle can be triggered by different factors. Recent studies showed that a mutual mechanism for muscle wasting results from the imbalance between protein synthesis and degradation, which are the mechanisms controlling anabolic/catabolic signals in a muscle cell. A deep look into the process of muscle atrophy revealed alterations of numerous molecular pathways participating in the management of muscle control. However, most of these pathways are still not fully understood [19,20]. Under physiological conditions, cooperation between different types of ncRNAs participates in the regulation of muscle differentiation and the management of protein synthesis and expression. So far, numerous miRNAs have been identified as key factors controlling protein function in a muscle cell; their alteration upon muscle atrophy was already confirmed [21]. Nevertheless, there is little known on the role of lncRNAs in myogenesis and muscle atrophy. Based on the function of known lncRNAs, they can either repress or activate gene expression through the regulation of gene transcription, mRNA stability, pre-mRNA splicing, miRNA sponging, protein translation, and protein stability. Until now, several studies mainly focused on the physiological function of lncRNAs in muscles, and the number of lncRNAs identified as regulators of muscle atrophy so far is still exiguous [22,23]. Therefore, the understanding of lncRNAs in muscle atrophy, mainly in inflammatory-induced muscle atrophy, is much more limited.

Collectively, very few lncRNAs, including MAR1 and lnc-mg as well as SMN-AS1, have been found to regulate muscle atrophy. The studies of myogenesis-related lncRNAs and the profiling of lncRNAs in muscle atrophy have shown the deserving hints for the investigation of lncRNAs in muscle atrophy. The knowledge on the putative function of lncRNAs in muscle atrophy is derived mainly from studies conducted on cell cultures and mice models [22]. Sun et al. identified a novel lncRNA, Atrolnc-1, in atrophying muscles from a mice model with cachexia. Interestingly, there was also an increase in the expression of Atrolnc-1 in the muscle of mice undergoing fasting and those with cancer or chronic kidney disease, suggesting different mechanisms of lncRNA activation. In a cultured C2C12 cell line, the overexpression of Atrolnc-1 increased protein degradation through interaction with ABIN1, resulting in enhanced NF-κB activity and MuRF-1 transcription [24]. The above mentioned results are partially in accordance with the study of Lei et al. In starvation-induced muscular atrophy conducted in vitro and in vivo, the authors also identified Atrolnc-1 as a factor affecting muscle wasting. Moreover, they identified a subset of novel lncRNAs mediating muscle wasting such as lnc-mg, lincMD1, Myolinc, lncMyoD, Dum, and MAR. However, their exact roles in the pathways mediating muscle wasting remain unidentified [25]. In another study, Hitachi et al. examined the expression of lncRNAs in six mice skeletal muscle atrophy models and cell lines (atrophic conditions: denervation, casting, tail suspension, dexamethasone administration, cancer cachexia, and fasting). They observed that the expression of DRR and DUM1 tended to be decreased in all models, and the expression levels of linc-YY1, Malat1, Neat1, and SRA showed significant changes in each model. However, they did not show consistent changes across the different muscle atrophy models [26]. In our study set, conducted on a C2C12 culture selectively affected by TNF-α to develop inflammatory-related muscle atrophy, we identified three lncRNAs (Gm4117, Ccdc41os1, and 5830418P13Rik) that are potentially involved in the mediation of inflammatory-related muscle loss. We also noticed a significant difference of dynamics changes in expression under different TNF-α concentrations and treatment times. Higher concentrations (>50 ng/mL) and longer treatment times (>24 h) with TNF-α affected the greater differences in lncRNAs’ expression between atrophy and control cells, suggesting their prospective role in the mediation of muscle wasting and the control of atrophy dynamics or severity. The conducted bioinformatics analysis provided data on their role in pathways related to muscle differentiation and loss. For instance, GO and KEGG enrichment analysis for Gm4117 revealed its role in the regulation of gene expression, RNA transport, and protein translation. Interestingly, we found that Gm4117 interacts with Npm1 and Mrpl42. It was demonstrated that Npm1 interacts with transcription factors, including c-Myc, NF-κB, YY1, and interferon regulatory factor 1 (IRF1) and is required for the regulation of their target genes. All of the aforementioned factors participate in the regulation of either muscle differentiation or inflammatory response. Hence, their downregulation can participate in the activation of muscle atrophy [27]. It was found that Mrpl42 interacts with the YY1 transcription factor, which enhances its activation. This observation could confirm the putative role in muscle cell management [28]. By the sponging of the miRNA-467/669 family, the Gm4117 is involved in the indirect regulation of FoxO and TNF signaling involved in muscle wasting. The miRNA-669 family was found to prevent skeletal muscle differentiation. Moreover, they are the first identified miRNAs that act upstream of MyoD, thus indirectly regulating all MyoD targets [29]. Ccdc41os1 can mediate muscle loss targeting the miRNA-125 family and, hence, participate in MAPK and FoxO signaling. The 5830418P13Rik regulates the protein catabolic process and response to cellular stress, which can modulate atrophy. The interaction of CCdc41os with kinase Cdk19 can modulate the inflammatory response in a muscle cell. It was proven that Cdk19 positively regulates inflammatory gene transcription in cooperation with NF-κB and C/EBPβ upon the stimulation of TLR9 [30]. Another target, Cgref1, modulates the activity of the AP-1 transcription factor that plays an important role in inflammation and muscle cell survival [31]. The miRNA-125b targeted by CCdc41os1 is involved in the regulation of the insulin-like growth factor signaling pathway and inhibits muscle cell differentiation and muscle regeneration and, thus, the mediation of muscle wasting [32]. The 5830418P13Rik, also known as Bfsp2-as in humans, was found deregulated in lung adenocarcinoma. This lncRNA exhibited significant associations with multiple immune cells, mainly T lymphocyte, neutrophil, and macrophage, implying Bfsp2-as may be an immune-related lncRNA in lung adenocarcinoma [33].

## 4. Materials and Methods

### 4.1. Cell Culture and Differentiation

The C2C12 mouse myoblast cell line was purchased from ATCC (American Type Culture Collection, Manassas, VA, USA) and cultured in Dulbecco’s Modified Eagle Medium (DMEM) (PAN-Biotech, Aidenbach, Bavaria, Germany) containing 10% fetal bovine serum (FBS) (PAN-Biotech, Aidenbach, Bavaria, Germany), 100 U/mL of penicillin, and 100 μg/mL of streptomycin at 37 °C in a humidified 5% CO_2_ and 95% air in incubator. Cells were plated onto plastic, noncoated culture flasks (for RNA extraction) or 8W10E plates for cell viability measurement in ECIS^®^ (Electric Cell-substrate Impedance Sensing). After reaching 80% confluence, DMEM with FBS was replaced by Differentiation Media (DM) containing 2% horse serum (PAN-Biotech, Aidenbach, Bavaria, Germany) and the abovementioned antibiotic mixture for 5 days to induce myoblast differentiation into myotubes. DM was changed every 48 h. After the differentiation. the cells in the atrophy model were treated with TNF-α and the control cells were treated with only free medium. Each study set was repeated in triplicate.

### 4.2. Atrophy Model

In the literature data, C2C12 myotube atrophy was established under different TNF-α concentrations and times of cell treatment. It mainly depended on cell confluence and the method on cell culturing: plates or flasks. Usually, the concentration of 10–100 ng/mL of TNF-α is used for atrophy model establishment with the treatment time ranging between 24 and 72 h. Prior to cells’ treatment with TNF-α, the myoblasts were cultured to about 80–90% confluence. In the discovery set, inflammatory-induced atrophy was performed by treating differentiated myotubes once with the addition of 100 ng/mL of mouse TNF-α recombinant protein (Thermo Fisher Scientific, Waltham, MA, USA) for 72 h. In the validation set, myotubes of the C2C12 cell line were treated with the following TNF-α concentrations: 10, 50, 100, and 200 ng/mL, respectively, for 72 h and under 100 ng/mL of TNF-α in 12- and 24-h time intervals, respectively, in order to monitor the dynamics in the lncRNA expression within the time interval and under different cytokine concentrations. The atrophy was established by a combination of cells’ degradation confirmed in microscopy examination, cell viability assay, and expression of muscle atrophy-related markers (Figure 1). Expression of Fbxo32 (atrogin-1) and MuRF1 (Trim63) as atrophy proof was measured by qPCR with fluorescently labeled probes (Thermo Fisher Scientific, Waltham, MA, USA). Then, expression was compared between the treated and untreated cells. The differences in the expression of atrophy markers between TNF-α-treated and untreated C2C12 cell cultures were calculated with the use of the Mann–Whitney U test. Each experiment was repeated in triplicate. The ECIS system Zθ instrument (Applied Biophysics Ltd., Troy, NJ, USA), supplied by ibidi GmbH (Gräfelfing, Germany), was used to measure the following electric parameters: impedance (Z), capacitance (C), and resistance (R). It consisted of two separate units: a station controller Zθ located outside the incubator and a docking station containing two eight-well plates, which was placed in the incubator space. In this study, we used 8W10E electrodes, which consisted of eight wells and 10 active electrodes in each well. The technique was applied to measure real-time cell parameters in the C2C12 cell culture under the TNF-α treatment and in the untreated/control C2C12 cells. After the stabilization process, the cells were seeded in plates at a density of ~1.2 × 10^5^ cells/mL per well and again placed in an incubator space under appropriate conditions. After 24 h, the medium was replaced by a differentiation medium containing 2% donor horse serum and treated under different concentrations of TNF-α. During the experiment, the cells’ survival was monitored for 72 h with ECIS software. The difference between the C2C12 cell cultures’ (treated and untreated) viability was compared using a one-way ANOVA test.

### 4.3. LncRNAs’ Profiling and Data Analysis

RNeasy Micro Kit (Qiagen, Toronto, ON, Canada) was used for total RNA purification from the harvested cells. Prior to the molecular experiments, all RNA samples were measured to determine the concentration, yield, and purity. Samples that passed quality measurements were diluted to 100 ng/mL each. A combination of lncRNA PCR Profiler Array Mouse Inflammatory Response, Autoimmunity, Cell Development, and Differentiation (Qiagen, Toronto, ON, Canada) allowed us to determine the expression in a total of 176 lncRNAs in a StepOnePlus device (Thermo Fisher Scientific, Waltham, MA, USA). Data analysis was based on the 2^−ΔΔCt^ algorithm with normalization of the raw data to housekeeping genes (set of 12 controls). All samples were analyzed in triplicates. A heat map of the lncRNA expression log values and the hierarchical clustering were conducted with the use of Morpheus software. To display statistical significance (*p*-value) versus fold change in order to identify key lncRNAs in the study set, the volcano plot was applied with the use of VolcaNoseR version2 software [34]. Expression differences of selected lncRNAs between treated and control samples were studied and visualized by MedCalc software (MedCalc, Ostend, Belgium). Chord diagrams with a hierarchical edge bundling were plotted to demonstrate a network of dependencies between differentially expressed lncRNAs (DElncRNAs) related to atrophy and physiological conditions. DElncRNAs were identified with *p* < 0.05 and fold log_2_ values < −2 and >2.

### 4.4. Dynamics in Expression Change of Selected lncRNA

The lncRNAs selected from the discovery set were independently validated by qRT-PCR in a StepOnePlus device (Thermo Fisher Scientific, Waltham, MA, USA) to measure changes in their expression in time intervals and under the different TNF-α concentrations. Total RNA was purified both from a set of cell cultures treated with concentrations of 10, 50, 100, and 200 ng/mL TNF-α, respectively for 72 h and from a set of cell cultures after 12-, 24-, 48-, and 72-h treatment with 100 ng/mL TNF-α, respectively. For each experiment, cell lines were cultured in triplicates. One-way ANOVA test was used to compare differences in lncRNAs’ expression between C2C12 cell cultures (treated and untreated).

### 4.5. Construction of DElncRNA Regulatory Network

Based on the mRNA–lncRNA and miRNA–lncRNA interactions, regulatory networks were searched with the available bioinformatics tools. IDs and sequences of previously selected mice DElncRNA were derived from RNAcentral and Ensembl databases. To predict the interaction between DElncRNA and mRNA, the ENCORI (the Encyclopedia of RNA Interactomes) and Mouse RGD tools were applied [35,36,37]. The sponging ability of DElncRNA for miRNA was predicted with the use of the following tools: ENCORI, Mouse RGD, miRWalk v2.0, DIANA-LncBase Predicted v2, and miRDB [38,39]. Venn diagrams were used to visually represent the similarities and differences between the miRNA predicted by the mentioned databases. The miRNA sequences demonstrating the highest degree of compliance among the databases were selected as key for pathway analysis. The Kyoto Encyclopedia of Genes and Genomes (KEGG) and miRPathv3.0 were used to explore and display molecular interactions of selected miRNA in signaling pathways [40,41].

### 4.6. Functional Annotation of Key Protein–Protein Network Construction

DElncRNA-mRNA regulatory networks were built, and the protein–protein interaction (PPI) network between them was constructed. The String v11.5 is a tool used for visualizing and searching for interactions between predicted and known proteins’ sequences [42]. Gene Ontology (GO) classification and KEGG pathway enrichment analysis were performed by using ShinyGO v0.75 software and String [43]. Results representing p values less than 0.05 were considered as statistically significant.

## 5. Conclusions

The knowledge of the role of lncRNAsin the mediation of muscle loss is still limited because of the different mechanisms that can trigger muscle atrophy. Perhaps different atrophic conditions can selectively alter individual cellular pathways and change the expression pattern of ncRNAs. We are aware that our study is not free from limitations. We used a single factor to develop atrophy; however, atrophic conditions develop usually upon action of various factors. We also reliedon in vitro results, and the findings of the experiments and the bioinformatics analysis should be confirmed by in vivo models. Moreover, results should be confirmed by siRNA/knockdown experiments. Nevertheless, in our study we demonstrated that muscle atrophy developing under inflammation mediated by TNF-α affects the alteration in lncRNAs’ machinery managing muscle conditions, and novel lncRNAs exclusive for mediation of inflammatory-related muscle loss were selected. This observation encourages the further investigation of lncRNAs in order to estimate their role in muscle atrophy and to broaden our knowledge of their role in the same.

## Data Availability

Not applicable.

## Supplementary Materials

The following supporting information can be downloaded at: https://www.mdpi.com/article/10.3390/ijms23073878/s1.
