# Supplementary material for: TNF-α Induced Myotube Atrophy in C2C12 Cell Line Uncovers Putative Inflammatory-Related lncRNAs Mediating Muscle Wasting
Source: Int J Mol Sci. 2022 Mar 31;23(7):3878. doi: 10.3390/ijms23073878 (PMC8998797; doi:10.3390/ijms23073878)
Supplement: Supplementary file 1 [file ijms-23-03878-s001.zip › ijms-1592690-supplementary.pdf]

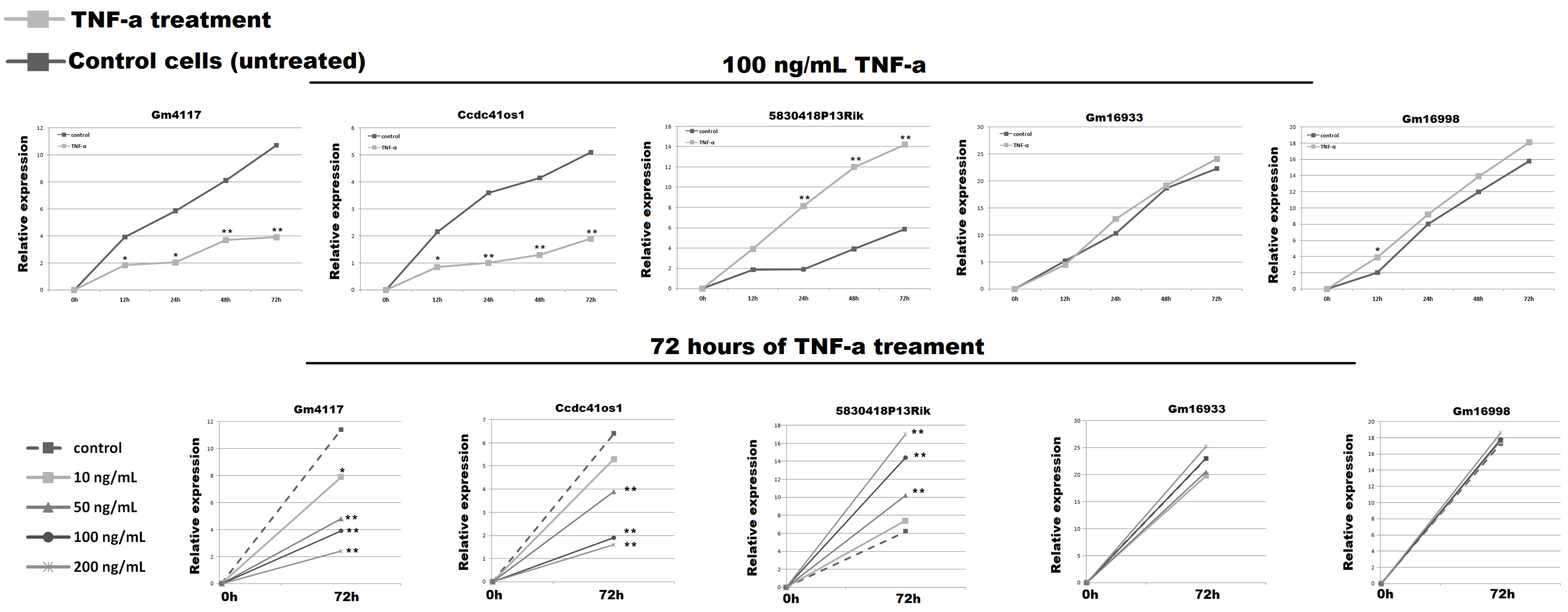

Supplementary Figure S1. Validation of the top 5 DElncRNAs in different TNF-α treatment conditions (*- p<0.05; ** - p<0.001)
